# Metabolic Phenotypes as Potential Biomarkers for Linking Gut Microbiome With Inflammatory Bowel Diseases

**DOI:** 10.3389/fmolb.2020.603740

**Published:** 2021-01-18

**Authors:** Stanislav N. Iablokov, Natalia S. Klimenko, Daria A. Efimova, Tatiana Shashkova, Pavel S. Novichkov, Dmitry A. Rodionov, Alexander V. Tyakht

**Affiliations:** ^1^A.A. Kharkevich Institute for Information Transmission Problems, Russian Academy of Sciences, Moscow, Russia; ^2^P.G. Demidov Yaroslavl State University, Yaroslavl, Russia; ^3^Atlas Biomed Group—Knomics LLC, London, United Kingdom; ^4^Center for Precision Genome Editing and Genetic Technologies for Biomedicine, Institute of Gene Biology, Russian Academy of Sciences, Moscow, Russia; ^5^Moscow Institute of Physics and Technology, Moscow, Russia; ^6^PhenoBiome Inc., San Francisco, CA, United States; ^7^Lawrence Berkeley National Lab, Berkeley, CA, United States; ^8^Sanford-Burnham-Prebys Medical Discovery Institute, La Jolla, CA, United States

**Keywords:** gut microbiome, metabolic phenotypes, inflammatory bowel diseases, machine learning, classifier, 16S rRNA sequencing

## Abstract

The gut microbiome is of utmost importance to human health. While a healthy microbiome can be represented by a variety of structures, its functional capacity appears to be more important. Gene content of the community can be assessed by “shotgun” metagenomics, but this approach is still too expensive. High-throughput amplicon-based surveys are a method of choice for large-scale surveys of links between microbiome, diseases, and diet, but the algorithms for predicting functional composition need to be improved to achieve good precision. Here we show how feature engineering based on microbial phenotypes, an advanced method for functional prediction from 16S rRNA sequencing data, improves identification of alterations of the gut microbiome linked to the disease. We processed a large collection of published gut microbial datasets of inflammatory bowel disease (IBD) patients to derive their community phenotype indices (CPI)—high-precision semiquantitative profiles aggregating metabolic potential of the community members based on genome-wide metabolic reconstructions. The list of selected metabolic functions included metabolism of short-chain fatty acids, vitamins, and carbohydrates. The machine-learning approach based on microbial phenotypes allows us to distinguish the microbiome profiles of healthy controls from patients with Crohn's disease and from ones with ulcerative colitis. The classifiers were comparable in quality to conventional taxonomy-based classifiers but provided new findings giving insights into possible mechanisms of pathogenesis. Feature-wise partial dependence plot (PDP) analysis of contribution to the classification result revealed a diversity of patterns. These observations suggest a constructive basis for defining functional homeostasis of the healthy human gut microbiome. The developed features are promising interpretable candidate biomarkers for assessing microbiome contribution to disease risk for the purposes of personalized medicine and clinical trials.

## Introduction

Recent advances in cultivation-based approaches for studying microbial diversity like culturomics (Bilen et al., 2018) allowed to isolate and characterize phenotypes and genomes of many human gut microbial species (Forster et al., 2019). High-throughput DNA sequencing of microbiome samples is still a method of choice for total profiling of the human microbiome. It provides semiquantitative taxonomic composition as well as functional potential, including biosynthesis of small molecules (Sugimoto et al., 2019). In the gut, the functional profile is generally less variable than the taxonomic one (Eng and Borenstein, 2018). Multiple species tend to be involved in complex ecological networks, many of which arise from cross-feeding, suggesting that the metabolic potential of a single species is less important than a community-wide gene-centric metabolism—provided the completeness of the pathways, however.

Deciphering the total microbiome network of metabolic interactions became possible with the advent of whole-genome shotgun (WGS) metagenomics. Even simple mapping of the reads to global reference gene catalogs already shows a strong variability between subjects both globally and by specific gene groups including carbohydrate catabolism, antibiotic resistance, and virulence factors (Yarygin et al., 2017), suggesting a more detailed investigation of distinct metabolic pathways. The extent of realization of the metabolic potential encoded in the microbiome can be evaluated quantitatively and qualitatively using metabolomics, particularly to elucidate its alterations linked to specific disorders. For example, targeted and untargeted metabolomic analysis of fecal samples from patients with inflammatory bowel diseases (IBD) revealed dysregulated metabolism of SCFAs, bile acids, tryptophan, and other molecules (Franzosa et al., 2019), suggesting that microbiota-derived metabolites play key roles in the pathogenesis (Lavelle and Sokol, 2020). To date, metabolomic experiments are more expensive and less standardized compared to high-throughput sequencing. The concept of predicting metabolite levels from metagenomic composition based on bacterial genome-scale metabolic models has shown promising results in the context of personalizing therapeutic dietary interventions for Crohn's disease (CD) patients (Bauer and Thiele, 2018).

The amplicon 16S rRNA sequencing is still the method of choice in terms of cost and information content for large-scale microbiome surveys of links between human microbiome and diet, diseases, verification of microbiome-related health claims of food products, and individual microbiome profiling. The sequenced variable 16S regions are often organized into operational taxonomic units (OTUs), i.e., clusters of similar sequences, or merged into the exact biological sequences present in the sample, so-called amplicon sequence variants (ASVs) that are further counted to get their relative abundances and taxonomically assigned using reference 16S databases (Prodan et al., 2020). Although this approach does not allow direct measurement of microbial gene content other than 16S rRNA itself, there are algorithmic methods for inferring the functional composition of the community from such data based on an *a priori* accumulated knowledge about microbial genomes (Aßhauer et al., 2015; Louca et al., 2016; Douglas et al., 2020; Narayan et al., 2020). Metagenomic prediction tools revealed functional alterations in the human gut microbiome linked to many diseases including IBD (Imhann et al., 2018), Parkinson's disease (Cirstea et al., 2020), and nonalcoholic fatty liver disease (Boursier et al., 2016), as well as to dietary interventions (Klimenko et al., 2018; Volokh et al., 2019) and other factors. However, the tools provide low accuracy for certain groups of functions. Firstly, some functional groups of genes are subject to frequent horizontal gene transfer across distant taxa (like antibiotic resistance determinants). Secondly, many metabolic pathways are established based on general databases and are neither curated to the point of sufficient accuracy nor take into account the specifics of a particular niche (like human gut). Meanwhile, precise metabolic reconstruction provides increased precision to elucidate ecological mechanisms of the communities (Sung et al., 2017; Garza et al., 2018).

Besides total analysis of all metabolic functions carried out by the gut microbes, some of them are often examined in a targeted manner as being highly relevant to the host–microbe interactions, ecological equilibrium, and diet, and of significant interest to be explored in novel datasets using interactive online systems (Efimova et al., 2018). The majority of these groups are short-chain fatty acid (SCFA) production, carbohydrate catabolism, and synthesis of vitamins and amino acids. The major SCFAs, namely, acetate, butyrate, and propionate, are synthesized by the gut microbes and are essential for host physiology by regulating inflammation, immunity, tumorigenesis, satiety, and involvement in signal functions (Koh et al., 2016). The propensity to synthesize them and the specific metabolic pathways vary across the bacterial kingdom, as showcased by butyrate (Vital et al., 2014). In the gut of IBD patients, there is a depletion of butyrate synthesis potential (Laserna-Mendieta et al., 2018). The main substrates for SCFA production are carbohydrates (glycans) of various structural complexities (oligo- and polysaccharides) coming as dietary fibers and general food components, making them the keystones in the prebiotic action of these molecules (Gibson et al., 2017). Different bacterial taxa have different capacities toward degrading a specific fiber type, and cross-feeding based on symbiotic catabolism of a complex glycan is not uncommon (Cockburn and Koropatkin, 2016; Cerqueira et al., 2020). Examination of an individual gut microbiome's total capacity for glycan catabolism suggests a way of designing personalized microbiome-tailored dietary plans (Klimenko et al., 2018).

Another prominent group of host health-relevant metabolites are vitamins. In the gut, the microbes can synthesize vitamin K and B vitamins along with their precursors (Rodionov et al., 2019). There are reports that at least some of the vitamins are accessible to the host (LeBlanc et al., 2013) and their fecal concentrations can be associated with clinical factors (McCann et al., 2019). Recent studies indicate that the importance of gut as a source of vitamins is limited and even a greater role of these vitamins might be in maintaining a robust ecological network between the species in the gut (Rodionov et al., 2019; Sharma et al., 2019). Interestingly, investigation of vitamin synthesis from stool metatranscriptomes of IBD patients showed an increased expression level of biotin (vitamin B7) biosynthetic enzymes compared to healthy controls (Das et al., 2019). Finally, amino acids released from undigested luminal proteins and peptides are accessible for gut microbiota that is involved in amino acid fermentation to form SCFAs and/or transformation to numerous metabolic end products such as phenols and indoles from aromatic amino acids (Davila et al., 2013). During intestinal inflammation, the microbiome potential for synthesis of amino acids, can decrease in favor of catabolism (Morgan et al., 2012). The therapeutic potential of amino acids for IBD has been proposed due to the reduction of inflammation, oxidative stress, and cell death in the gut they can evoke (Liu et al., 2017).

Development of high-precision and accuracy approaches for profiling of these selected functions will provide a valuable tool for efficient mining of biomedically relevant information from amplicon sequencing data and improving the downstream interpretations of gut microbiome data. Previously, we developed a new genomics-based methodology of predictive phenotype profiling that computes CPI (Community Phenotype Index) values as community-wide fractional representation of a limited set of basic metabolic phenotypes (such as amino acid auxotrophy and sugar utilization capabilities) deduced from *in silico* reconstruction over a large reference collection of HGM genomes and projected over 16S rRNA abundance profiles of the analyzed samples (Rodionov et al., 2019). This predictive metabolic phenotype profiling methodology was further applied to characterize the 16S rRNA amplicon-based taxonomic profiles of the fecal microbiomes in *in vivo* and *in vitro* studies and identify metabolic phenotypes that are linked to variable diets or growth conditions (Peterson et al., 2019; Sharma et al., 2019; Elmén et al., 2020; Jones et al., 2020). Here we used this *in silico* metabolic phenotype profiling approach to identify the links between the functional homeostasis of gut microbiome and disease. To assess the performance of our approach in discovering novel robust biomarkers, we applied it to the case of the IBD that are associated with the altered microbiome composition, along with the genetic, lifestyle and environmental factors (Beaugerie et al., 2018).

## Materials and Methods

### Study Design and Raw Sequence Data Analysis

For our analysis, we selected the following three previously published IBD datasets with publicly available 16S rRNA gene sequencing data of stool samples. The Spanish dataset (ESP) included 34 Crohn's disease (CD) patients, 33 ulcerative colitis (UC) patients, and 101 healthy controls (HC) (Pascal et al., 2017). The Chinese dataset (CHN) included 72 CD, 51 UC, and 71 healthy individuals (Zhou et al., 2018); an additional 16 CD patients on infliximab treatment were excluded from the analysis. Both ESP and CHN datasets were further filtered to retain only one sample per individual in cases of multiple replicates. The study of 313 IBD patients from the Netherlands (NLD) included 188 patients with CD, 107 patients with UC, and 18 additional IBD patients with either intermediate or undetermined disease status (Imhann et al., 2018); the latter were excluded from the study. The healthy NLD group originally included 1,010 individuals from the LifeLines DEEP cross-sectional general population study (Tigchelaar et al., 2015) that was further reduced to 495 healthy controls selected as previously described in (Imhann et al., 2018).

The raw sequence data from the CHN and ESP datasets were downloaded from the European Nucleotide Archive (www.ebi.ac.uk/ena)—project IDs PRJNA422193 and PRJEB22028, respectively. The NLD datasets were obtained from the European Phenome-Genome Archive (https://ega-archive.org/), project IDs EGAS00001002702 and EGAS00001001704. The 16S rRNA gene sequences (hypervariable region V4) were analyzed using the DADA2 plugin from the QIIME2 package (Callahan et al., 2016; Bokulich et al., 2018). Briefly, raw demultiplexed reads were quality filtered, denoised, and dereplicated into ASVs and the read counts (relative abundance values) were calculated for each ASV in each sample. Average abundance loss after DADA2 filtering was 23% for ESP, 27% for CHN, and 12% for NLD datasets. The obtained ASV abundance tables were additionally filtered to retain only the amplicons satisfying at least one of the following criteria: (i) ASV is present in >0.5% of samples; (ii) dataset-average ASV abundance >0.25%; and (iii) maximum ASV abundance >0.5% across the dataset. As a result, each of the three datasets were filtered with 1–2% average abundance loss per sample. Finally, we filtered each dataset by a minimal coverage (number of reads per sample). For ESP and NLD, the coverage threshold was >15,000 reads, whereas for the CHN, it was set to >4,000 reads due to overall low read counts for this dataset. The distributions of read numbers are shown in Supplementary Figure 1. The numbers of samples retained for further analysis in the three datasets are provided in Table 1.

**Table 1:** Number of samples per dataset and per clinical status analyzed in this work.

**IBD status/dataset**	**Spain (ESP)**	**Netherlands (NLD)**	**China (CHN)**
Healthy controls (HC)	91	496	67
Crohn's disease (CD)	34	163	50
Ulcerative colitis (UC)	39	99	37

### Taxonomic Assignment

Taxonomic classification of ASV representative sequences was performed using the multi-taxonomic assignments (MTA) approach as described below. Each representative sequence was aligned using NCBI BLAST ToolKit against a joined reference 16S rRNA database including sequences from RDP database version 11.5 (Cole et al., 2014) and NCBI 16S database version of December 2019. Alignment results were sorted according to their identity *F* (as a fraction of 1), with the maximum *F*-value for the best hit denoted as *M*. Alignment hits with value of *F* in the range between *M* and *M*–(1–*M*)/*S* and greater than a threshold value *D* were selected for MTA. Here, *S* acts as a scaling parameter, which controls the list of taxonomic descriptions accepted for MTA based on the *F* value of the alignment and was taken equal to 4. The drop threshold parameter *D* was taken equal to 0.85. The resulting MTA for each ASV represented a list of unique regular taxonomic descriptions with equal weights assigned to each item. String representations for MTA consisted of slash-separated names of taxa on each taxonomic level. The main advantage of the MTA over the consensus-based methods consists in its ability to assign taxonomic descriptions up to the genus (and even species) level for sequences with low identity and, hence, poor genus-level resolution. In this case, the organisms with partially resolved genus-level taxonomies could also participate in machine learning, providing multi-taxonomic descriptions as features. However, the broader the list of accepted taxonomies for MTA is, the less useful the corresponding feature becomes for cross-study analysis. For example, multi-taxonomic descriptions A/B/C for an ASV in one study and A/B/D for a closely related ASV in another study are considered as different features. To increase the overlap in the sets of taxonomic features between different studies, one should aim for shorter MTA strings. This motivates the strict choice of S and D parameters, which leads to compact MTA descriptions.

### Prediction of Metabolic Phenotypes in Reference Genomes

Functional gene assignments and metabolic reconstructions were performed using the SEED database/tools that allow a subsystem-based analysis of ~6,000 bacterial genomes, including a subset of 2,662 reference human gut microbial (HGM) genomes representing 690 individual species (Overbeek et al., 2014). The subsystem-based approach for metabolic reconstruction combines protein similarity search, analysis of chromosomal gene clusters, and phylogenetic profiling (Overbeek et al., 2005). The collection of curated subsystems includes metabolic pathways for (i) biosynthesis of essential nutrients (vitamins, amino acids); (ii) uptake and fermentation of carbohydrates including mono-, oligo-saccharides, sugar acids, and alcohols; (iii) degradation of amino acids; and (iv) production of SCFAs. The metabolic subsystems were developed based on the previously published genomic studies of phylogenetic distribution of bacterial pathways for metabolism of vitamins and amino acids, utilization of carbohydrates, and production of butyrate and propionate in HGM bacteria (Rodionov et al., 2011, 2013, 2019; Ravcheev et al., 2013; Khoroshkin et al., 2016; Leyn et al., 2017; Arzamasov et al., 2018; Bouvier et al., 2019; Feng et al., 2020). Using the collection of pathway-specific logical rules (Rodionov et al., 2019), we obtained Binary Phenotype Matrix (BPM) describing 94 inferred phenotypic features (nutrient requirements, utilization capabilities, metabolite production) of each reference genome as binary (“1” or “0”) phenotypes and reflecting the presence/absence of a complete catabolic or biosynthetic pathway. In addition to catabolic enzymes, the sugar utilization subsystems also included sugar-specific uptake transporters; thus, the assigned sugar utilization capability required the presence of both catabolic pathway and uptake transporter. We also obtained the distribution of 229 families of glycosyl hydrolases (GHs) in the analyzed reference genomes using dbCAN2 tool (Zhang et al., 2018). The obtained GH family distribution was converted to GH-BPM, where each column represents an individual GH family, and each binary phenotype represents the presence or absence of at least one enzyme from this family. The obtained metabolic BPM and GH-BPM for 2,662 reference genomes provided as a part the Phenotype Profiler tool (see below) were used to calculate the Community Phenotype Index (CPI) for each metabolic phenotype or GH family and each 16S rRNA sample as previously described (Rodionov et al., 2019) and explained in more details below.

### Calculation of the Metabolic Phenotype Profile

To obtain phenotype profiles for analyzed 16S rRNA samples, we utilized the Phenotype Profiler tool provided by PhenoBiome Inc. (San Francisco, CA). First, we mapped each ASV obtained from the samples to a reference collection of 2,662 microbial genomes based on their 16S rRNA gene sequence match. The reference HGM genome collection was analyzed with Barrnap (https://github.com/tseemann/barrnap) to predict the location of ribosomal RNA genes and select all 16S rRNA gene sequences for each genome. Partial 16S rRNA gene sequences were replaced with corresponding complete sequences from the NCBI 16S rRNA database. In order to establish such mapping, each ASV sequence was first aligned against the reference 16S rRNA collection using the NCBI BLAST standalone toolkit. To further assign reference organisms to ASV, we used the same top hit selection criteria as in the MTA procedure with the same values for S and D as described above. The reference organisms corresponding to the selected alignment hits, therefore, constituted a mapping for each ASV with weights distributed equally.

To calculate CPI values for each sample and for each metabolic phenotype, we first obtained a probabilistic estimate *p*_*i*_ for a given ASV (that corresponds to one or more reference genomes) to possess a certain binary metabolic phenotype as a mapping-weighted average across BPM. CPI values were calculated as:

$$\begin{array}{l} CPI=\underset{i}{\sum }p_{i}A_{i} \end{array}$$

where the sum is taken over all ASVs and *A*_*i*_ represents a particular ASV's relative abundance. CPI provides a fractional representation of cells in the community possessing a specific metabolic pathway or GH family.

Phenotype alpha diversity (PAD) metric was calculated for each metabolic phenotype for each sample as an alpha diversity of microbial ASVs possessing a particular phenotype. In order to do this, we first aligned ASVs' representative sequences using MUSCLE (Edgar, 2004). Next, an unrooted tree was built from the alignment using FastTree 2 (Price et al., 2010). Finally, the tree was rooted according to the midpoint strategy and used to compute Faith's phylogenetic alpha diversity metric with the Python scikit-bio (http://scikit-bio.org) package. For each sample and each phenotype, only those ASVs that had map-averaged expected phenotype values >0.6 were considered as the ones having the potential to express certain phenotypes.

### Machine Learning for Clinical Status Prediction

#### Classification Setups

To construct the microbiome-based classifiers of clinical status (HC, CD, or UC), we used the taxonomic and metabolic phenotype profiles obtained for 3 datasets (CHN, ESP, NLD) as input features for Random Forest classifiers (implemented in Python's scikit-learn RandomForestClassifier). The classifiers were different by three strategies, two sets of predictors, and two IBD clinical states (CD or UC). We used the following strategies: (i) classification of each dataset separately using two-thirds of samples as a training set and one-third as a testing set (Single strategy); (ii) classification of a mixed dataset constructed from an equal number of healthy and randomly selected affected subjects from every dataset using two-thirds as a training set and one-third as a testing set (Mixed strategy); and (iii) classification of joined datasets constructed by combining each dataset pair as a training set and the remaining third dataset as a testing set (leave-one-out strategy, L1O). By applying these strategies to the three analyzed datasets (CHN, ESP, NLD), we obtained seven variants of classifiers (including three variants for the L1O strategy, three variants for the Single strategy, and one variant for the Mixed strategy). We also considered two different sets of predictors including (i) taxonomic names at the genus level and their corresponding abundances and (ii) metabolic phenotypes and their corresponding CPI values, and two IBD clinical states CD and UC) compared with healthy controls (HC). As a result, we designed 28 types of RF classifiers.

The following parameters were used to build each RF classifier:

1. tree depth = 3;
2. number of trees = 200;
3. percent of features in each split = 50%;
4. balanced class weights.

The use of balanced class weights for cost function calculation ensured that classes (clinical status) were weighted inversely proportional to their frequency in the training set.

For the Single and Mixed strategies, we performed random subsampling and 10 cross-validation iterations to evaluate mean performance characteristics. In the L1O strategy, two combined datasets served as a training set with the remaining one being a testing set; thus, we performed three cross-validation iterations without random subsampling.

#### Feature Filtration and Extraction

**Figure 1:**
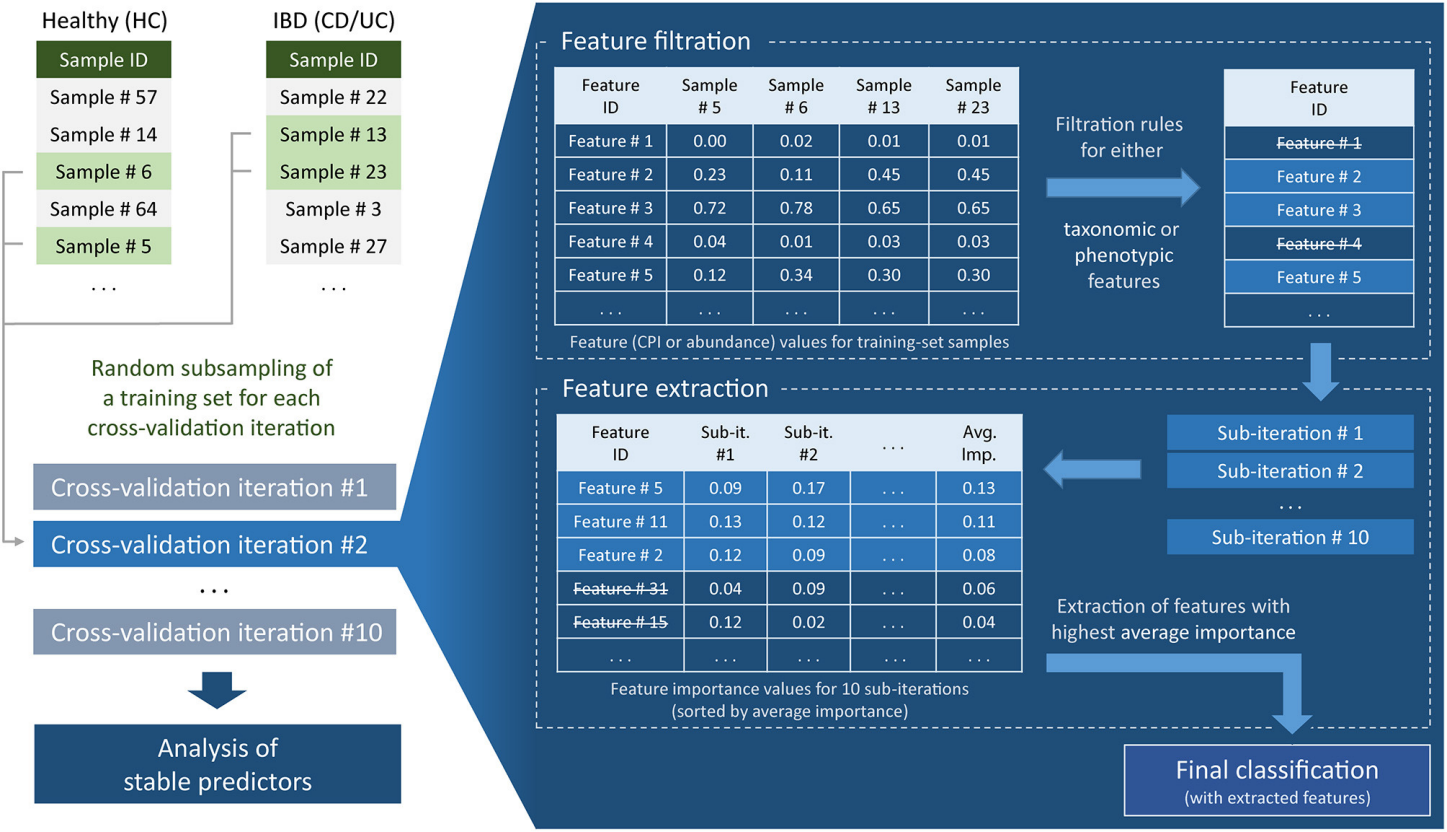
Flowchart of machine learning approach for clinical status prediction based on microbiome data.

For each cross-validation iteration, we also performed feature filtration and feature extraction (Figure 1). These procedures were based only on the information contained in the training part of the data. Feature filtration was done according to the following sets of rules, for taxonomic as well as for the phenotypic features. For taxonomic features, the taxa satisfying at least one of the following criteria were filtered out: (i) nonzero abundance in <5 samples and (ii) maximum abundance across the training set <1%. For phenotypic features, phenotypes satisfying at least one of the following criteria were filtered out: (i) mean CPI value out of the range of [0.1, 0.9]; (ii) CPI value >0.05 in <5 samples; and (iii) mean PAD value <3.5. For the strategies including more than one dataset (MC, L1O), all above filtering conditions were required to be satisfied in each dataset separately. Feature filtration was followed by the feature extraction including 10 sub-iterations of classification with the same classifier parameters as before. After each sub-iteration, we evaluated feature importance (decrease of Gini impurity) based on the training set data. The top 20 features with the highest mean importance values were used in the final classification.

### Subsampling

The groups of samples were subsampled for each of the aforementioned classification strategies except for the Single strategy in order to equalize the contribution of each dataset to the training set. For the Mixed strategy, this resulted in the following numbers of samples per IBD clinical status in each dataset: 67 for HC, 34 for CD, and 37 for UC. For the L1O strategy, these numbers were dependent on a particular strategy variant (Table 2). The disproportion in the number of samples between the CD/UC/HC classification groups was accounted for by using balanced class weights.

**Table 2:** Number of samples per dataset and per classification group used for the L1O classification strategy.

**Strategy**	**L1O:ESP**			**L1O:NLD**			**L1O:CHN**		
**Description**	**CHN+NLD (training)**			**CHN+ESP (training)**			**NLD+ESP (training)**		
	**ESP (testing)**			**NLD (testing)**			**CHN (testing)**		
**Dataset**	**CHN**	**NLD**	**ESP**	**CHN**	**ESP**	**NLD**	**NLD**	**ESP**	**CHN**
HC	67	67	91	67	67	496	91	91	67
CD	50	50	34	34	34	163	34	34	50
UC	37	37	39	37	37	99	39	39	37

#### Performance Evaluation

To assess classification quality, we conducted the ROC curve analysis on each cross-validation iteration and calculated the following metrics: Area Under the Curve (AUC), sensitivity, and specificity. Then, for each of 28 classifier types, we estimated mean values and corresponding standard deviations of these metrics.

#### Analysis of Stable Predictors

We identified a collection of stable predictors (phenotypes or genera) for each disease status using feature importance analysis. For each classifier type, mean feature importances (mean decrease of Gini impurity) were calculated across 10 cross-validation iterations. If some feature was not extracted (during filtration and extraction procedure) in one or more cross-validation iterations, then its importance value was set to zero. A predictor was defined as stable for a given disease status if it satisfied the following criteria: (i) nonzero importance values in at least six out of seven classifier types corresponding to possible combinations of classification strategy and dataset and (ii) the same-sign difference between mean predictor values (CPI or abundance) for HC and CD/UC groups in each dataset. The importance for each stable predictor was taken to be its average value across possible combinations of classification strategy and dataset.

To investigate the relationship between stable predictors and clinical status, we constructed additional classifiers based only on stable predictors for the Mixed classification strategy for each combination of predictor set and disease status. For each such classifier, 20 cross-validation iterations were performed. We also constructed single-feature partial dependence plots (PDP) in each iteration for each of the stable predictors with resolution of 20 grid steps and probability of CD or UC outcome as a response. The mean dependency was calculated across the iterations. Based on the form of the dependency, the predictors were classified into five categories—sharply decreasing, sharply increasing, smoothly decreasing, smoothly increasing, and unclassified. The classification into categories was performed by analyzing the differences of probability values (Δ*i*) between the (*i*+5)-th and *i*-th step (for each *i* from 1 to 15) in the following way. First, the direction of dependence (increasing or decreasing) was determined by the sign of Δ_*i*_ with the highest absolute value: positive sign corresponds to the increasing PDP and negative—for decreasing. If one predictor had at the same time positive and negative Δ_*i*_, and the highest ratio between their absolute values was <2, then the predictor was defined as unclassified. Second, the form of dependence (sharp or smooth) was determined by the following rule: if at least one of the absolute Δ_*i*_ values was >= 3 times higher than the maximum probability differences calculated for outer lower [1, *i*) and upper (*i*+5,20] intervals, then the sharp form was chosen. Otherwise a smooth form was chosen.

## Results

### Predicted Metabolic Phenotype and Taxonomy Profiles of IBD Samples

We selected three previously published fecal microbiome datasets representing geographically distinct cohorts of IBD patients from China (CHN) (Zhou et al., 2018), Spain (ESP) (Pascal et al., 2017), and the Netherlands (NLD) (Imhann et al., 2018). Each analyzed dataset included two groups of IBD subjects with either Crohn's disease (CD) or ulcerative colitis (UC) diagnosis and also a group of healthy control (HC) subjects from the same geographical population. Having filtered the three datasets to remove duplicate samples representing the same subject and samples from subjects that were treated with immunosuppressants, we have analyzed raw 16S rRNA amplicon sequence data for each dataset individually. We applied QIIME2's DADA2 plugin to obtain the ASV abundance profiles and then filtered out the samples with low read counts as described in section Materials and Methods. The number of remaining samples per dataset and per clinical status group is provided in Table 1.

**Figure 2:**
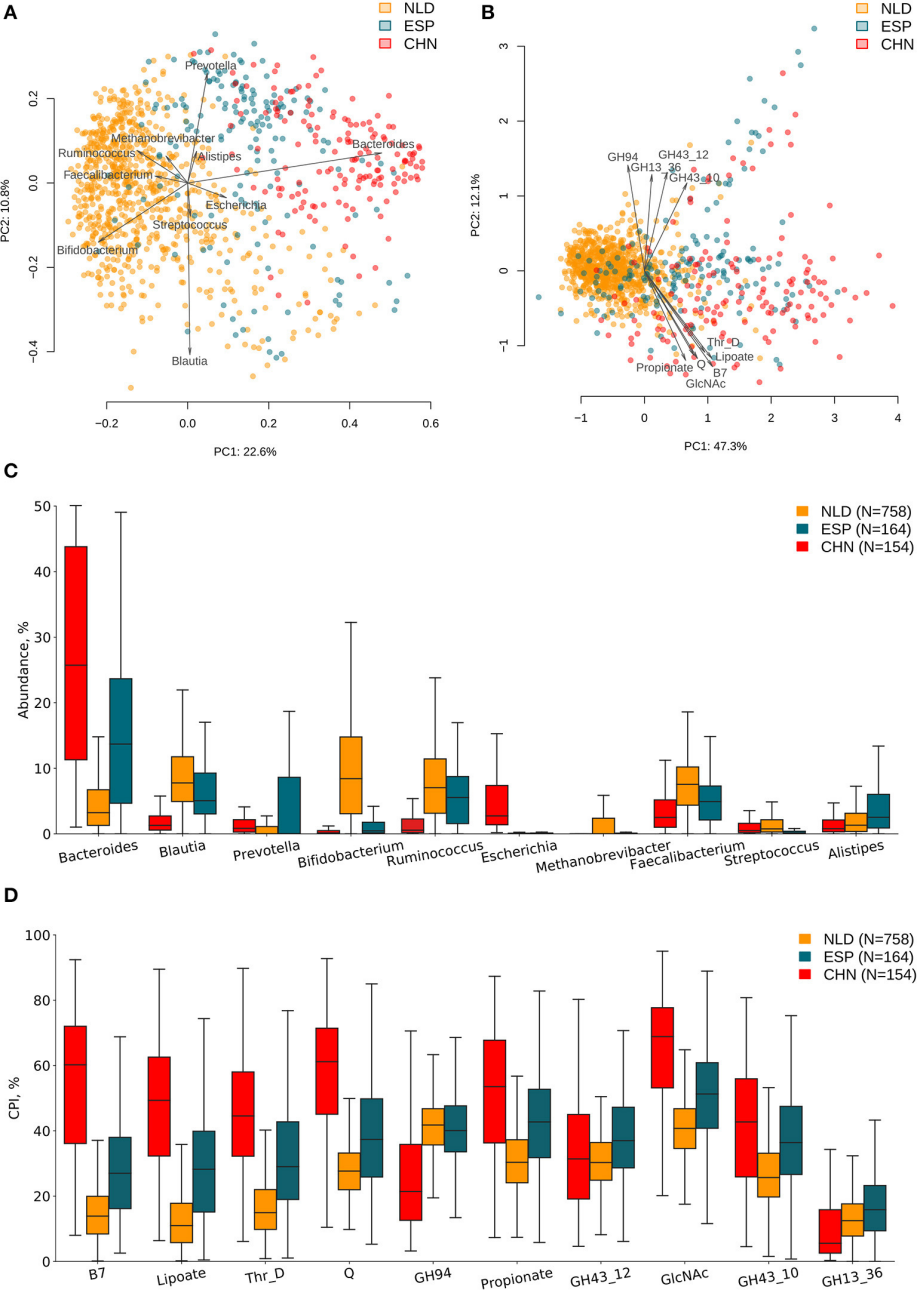
Differences in microbiome composition and metabolic functions of samples from three investigated IBD datasets. **(A)** PCoA analysis of samples using taxonomic features (genera abundance) and Bray–Curtis dissimilarity metric for distance calculation. **(B)** PCA analysis of samples using metabolic phenotype features (CPI) for distance calculation. Arrows show top 10 features in terms of the explained variance in the given axes. Arrow length is proportional to the percent of variance explained by the feature. Distribution of taxonomic abundances **(C)** and Community Phenotype Index (CPI) values **(D)** for the corresponding top 10 features across three IBD datasets.

ASVs' taxonomies were obtained using the multi-taxonomic assignment (MTA) approach. The analysis of abundance distribution for the top 20 taxonomic genera demonstrated a much larger variability between the analyzed three datasets and moderate variations between groups of samples with a different clinical status within each dataset. Further, we analyzed the obtained ASV profiles using the metabolic phenotype profiling approach (Rodionov et al., 2019) to calculate the sample-wise Community Phenotype Indices (CPIs) for 94 metabolic phenotypes from BPM constructed from the reference collection of 2,662 HGM genomes. Using the same approach, we calculated CPI values for 229 GH families from GH-BPM constructed from the genomic distribution of GH enzymes in reference genomes (see section Materials and Methods). Additionally, for each phenotype (and GH family), we estimated the corresponding Phenotype Alpha Diversity (PAD) values, which was defined as the phylogenetic alpha diversity of a subcommunity of corresponding phenotype carrier (as described in section Materials and Methods). The obtained CPI and PAD values for the analyzed metabolic phenotypes and GH families across all samples in the three analyzed datasets are shown in Supplementary Tables 1, 2, while the genus-level taxonomic profiles are presented in Supplementary Table 3. We performed principal component analysis (PCA) of the phenotypic features and principal coordinate analysis (PCoA) of the taxonomic features calculated for samples from the three IBD datasets and revealed visible differences between microbiome composition of samples from each of these datasets (Figures 2A,B). Distributions of top 10 variable features among taxonomic genera and phenotypic CPIs across the three IBD datasets are provided in Figures 2C,D. Among the most variable taxonomic genera are *Bacteroides* (in CHN and ESP datasets), *Blautia* (in NLD and ESP), and *Bifidobacterium* (in NLD), while the list of most variable metabolic phenotypes is largest in the CHN dataset and includes biosynthesis of vitamins (biotin/B7, queuosine/Q, lipoate) and propionate, utilization of N-acetylglucosamine (GlcNAc), degradation of threonine (Thr_D), and the presence of specific GH families (GH94, GH43_12, GH43_10, GH13).

### Classification of IBD Status Based on Taxonomy and Microbial Phenotypes

The obtained metabolic phenotype profiles (CPI and PAD values) and genus-level taxonomic profiles were used to examine the potential of microbiome features to predict IBD clinical status using the Random Forest (RF) classification approach. As an input for the RF classifier, we used two types of features, either relative taxonomic abundances at the genus level or CPI values for predicted metabolic phenotypes. Both types of features were filtered during the cross-validation procedure according to a set of rules (see sections Materials and Methods, Figure 1). Particularly, the corresponding PAD values served as one of the filtering criteria to eliminate phenotypes with contribution to CPI coming from a phylogenetically narrow group of organisms. The IBD clinical status (CD, or UC and HC) was used as the RF classifier output. For each of the three analyzed datasets (NLD, ESP, CHN), we applied three division strategies (Single, Mixed, and L1O, see section Materials and Methods) to obtain seven different variants of division of the analyzed datasets on training and testing sets. In total, we obtained 28 classifiers for two pairs of IBD clinical states (CD vs. HC, and UC vs. HC), two sets of predictors (taxonomies or phenotypes) and three division strategies.

#### Crohn's Disease Classifiers (CD-vs.-HC)

**Figure 3:**
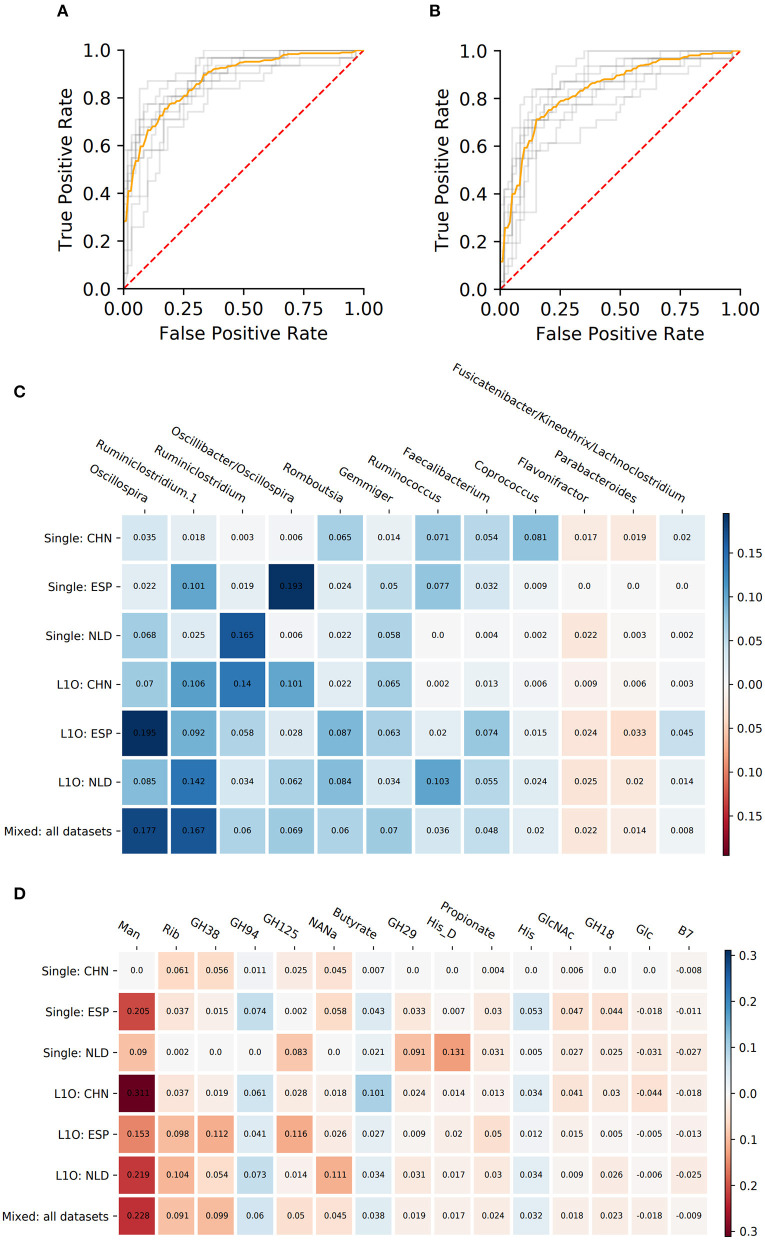
Performance characteristics and stable predictors for the CD-vs.-HC classifier. **(A,B)** ROC curves for the Mixed strategy for CD-vs.-HC classifiers with taxonomic **(A)** and phenotypic **(B)** predictors. **(C,D)** Stable predictor importance values in different classification variants for taxonomic **(C)** and phenotypic **(D)** predictors. Color saturation corresponds to the mean importance of the predictor (see color key). Color hue corresponds to the direction of the difference between HC and CD means (blue—increased in HC, red—increased in CD).

As a result, we obtained 14 CD-vs.-HC classifiers, with corresponding performance characteristics listed in Table 3. In general, the taxonomy-based classifiers demonstrated higher AUC and sensitivity values than the phenotypes-based classifiers (Figures 3A,B). On the contrary, the latter showed higher specificity values. By comparing features that were selected after feature filtration and feature extraction steps in different strategies (see Figure 1 and section Materials and Methods for more details), we extracted 15 phenotypic and 12 taxonomic features that work as stable predictors (Figures 3C,D). The selected taxonomic groups and phenotypes demonstrated variable average importance values across different classification strategies and datasets. For the majority of stable taxonomic predictors (10 out of 12 taxa), mean abundance values were higher in healthy controls than in CD patients. However, for the majority of phenotypic predictors (12 out of 15 phenotypes), mean CPI values were lower in healthy controls than in CD patients.

**Table 3:** Mean performance characteristics of the taxonomy- and phenotype-based CD classifiers over 10 classification iterations.

**Strategy/dataset**	**Taxonomy-based classifier**			**Phenotypes-based classifier**		
	**Mean sensitivity**	**Mean specificity**	**Mean AUC**	**Mean sensitivity**	**Mean specificity**	**Mean AUC**
Single:CHN	0.7867	0.7857	0.8463	0.7333	0.7571	0.8463
Single:ESP	0.7900	0.9179	0.9511	0.7700	0.9036	0.915
Single:NLD	0.7755	0.9161	0.9344	0.6918	0.9020	0.8920
L1O:CHN	0.9440	0.4358	0.7985	0.850	0.6164	0.7725
L1O:ESP	0.9353	0.8165	0.9484	0.8176	0.8516	0.8895
L1O:NLD	0.4252	0.9871	0.8567	0.3534	0.9899	0.7632
Mixed: all datasets	0.7548	0.8083	0.8736	0.7065	0.8350	0.8339
Mean across all variants	0.7731	0.8096	0.8870	0.7032	0.8365	0.8446

*In the L1O strategy description, the name of the dataset corresponds to the testing dataset (for example, the “L1O:CHN” description means that the classifier was trained on the ESP and NLD datasets and tested on the CHN dataset). Cell color reflects the characteristics' values (greater values correspond to darker colors)*.

**Figure 4:**
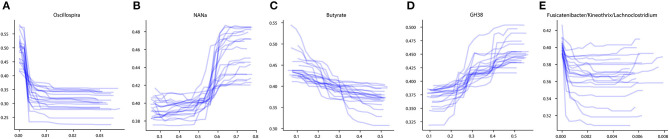
Examples of different PDP forms. **(A)** Sharply decreasing PDP. **(B)** Sharply increasing PDP. **(C)** Smoothly decreasing PDP. **(D)** Smoothly increasing PDP. **(E)** Unclassified PDP. The *x*-axis of the plots denotes the taxonomic or phenotypic feature abundance, and the *y*-axis shows the probability of the CD classification outcome.

To investigate the influence of each stable predictor on the classification result, the partial dependence plot (PDP) analysis was performed. Using the Mixed strategy, we constructed taxonomy-based and phenotype-based classifiers, with only stable predictors as input features. For each stable predictor, a PDP was obtained, and the predictors were classified based on their PDP form into five categories: sharply decreasing, sharply increasing, smoothly decreasing, smoothly increasing, and unclassified (see section Materials and Methods). In these descriptions, the term “increasing” means that the probability of CD outcome is greater for greater predictor values, and vice versa for the term “decreasing.” The examples of PDP forms are shown in Figure 4. The grouping of predictors into PDP form categories is listed in Table 4, and all PDPs are shown in Supplementary Figures 2, 3.

**Table 4:** The forms of stable predictors PDPs (term “increasing” means increasing probability of the disease).

**PDP form type**	**CD (taxonomy; phenotypes)**	**UC (taxonomy; phenotypes)**
Sharply decreasing PDP	*Ruminiclostridium.1, Oscillospira, Oscillibacter/Oscillospira, Gemmiger, Romboutsia, Ruminococcus, Ruminiclostridium, Coprococcus, Fusicatenibacter/Kineothrix/ Lachnoclostridium, Faecalibacterium*, GH94, His	G*emmiger, Ruminococcus, Akkermansia*; –
Smoothly decreasing PDP	Butyrate	–; His, Arg
Sharply increasing PDP	NANa, Rib, GH18	–; –
Smoothly increasing PDP	*Flavonifractor*; Man, GH125, GH38, GlcNAc, GH29, Propionate, Glc, His_D, B7	*Streptococcus, Oscillibacter, Flavonifractor, Flintibacter;* Man, GH125
Unclassified	*Parabacteroides*	–; –

The majority of stable taxonomic predictors (10 out of 12 taxa) for CD-vs-HC classifiers demonstrated sharply decreasing PDP forms (Supplementary Figure 2, Table 4). Moreover, for each of them, the abundance threshold for the sharp decrease of CD output probability was close to zero (e.g., for *Oscillospira*, see Supplementary Figure 2). It suggests that the probability of CD as an outcome was high in the complete absence of the corresponding taxon in the community and dropped sharply even with the small increase of its abundance; the further increase of the abundance did not considerably affect the output. Noteworthily, the majority of phenotypic predictors (12 out of 15) had an increasing PDP form, smooth or sharp (Table 4, Supplementary Figure 3).

#### Ulcerative Colitis Classifiers (UC-vs.-CD)

**Figure 5:**
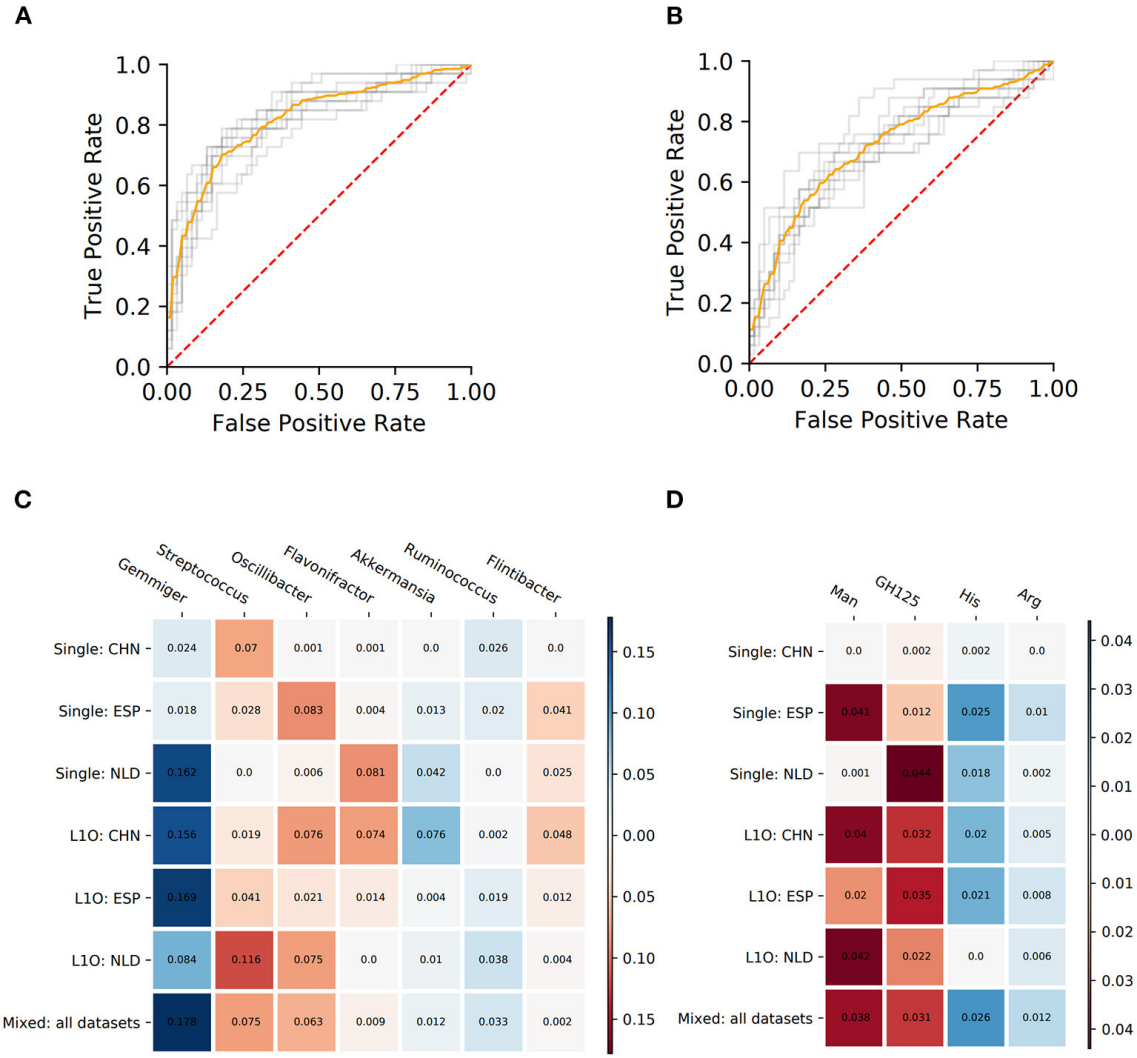
Performance characteristics and stable predictors for the UC-vs.-HC classifier. **(A,B)** ROC curves for the Mixed strategy for UC-vs.-HC classifier construction with taxonomic **(A)** and phenotypic **(B)** predictors. **(C,D)** Stable predictor importance values in different classification variants for taxonomic **(C)** and phenotypic **(D)** predictors. Color saturation corresponds to the mean importance of the predictor (see color key). Color hue corresponds to the direction of the difference between HC and UC means (blue—increased in HC, red—increased in UC).

Similar procedures were performed to obtain 14 UC-vs.-HC classifiers. Their performance characteristics are listed in Table 5. In general, taxonomy-based classifiers demonstrated higher AUC, sensitivity, and specificity values than phenotype-based classifiers (Figures 5A,B). For the L1O:NLD classification variant, AUC values for the phenotype-based classifier (0.43) was close to that of a random guess (0.5). Overall, all constructed UC-vs.-HC classifiers demonstrated lower performance characteristics when compared to the corresponding CD-vs.-HC classifiers.

**Table 5:** Mean performance characteristics of the taxonomy- and phenotype-based UC classifiers over 10 classification iterations.

**Strategy: dataset**	**Taxonomy-based classifier**			**Phenotype-based classifier**		
	**Mean sensitivity**	**Mean specificity**	**Mean AUC**	**Mean sensitivity**	**Mean specificity**	**Mean AUC**
Single:CHN	0.7909	0.9048	0.9277	0.8091	0.8857	0.9281
Single:ESP	0.3000	0.8667	0.6583	0.3250	0.7741	0.5815
Single:NLD	0.6300	0.9376	0.9240	0.5500	0.8678	0.8232
L1O:CHN	0.6324	0.5955	0.6791	0.4189	0.8791	0.6832
L1O:ESP	0.3051	0.8626	0.6607	0.3308	0.7714	0.5751
L1O:NLD	0.7354	0.4972	0.6944	0.8364	0.1022	0.4341
Mixed: all datasets	0.5970	0.8590	0.8153	0.5667	0.7836	0.7240
Mean across all variants	0.5701	0.7891	0.7656	0.5481	0.7234	0.6785

*In the L1O strategy description, the name of the dataset corresponds to the test set (for example, the “L1O:CHN” description means that the classifier was trained on the ESP and NLD datasets and tested on the CHN dataset). Cell color reflects the characteristics' values (greater values correspond to darker colors)*.

Despite the fact that in some strategies the corresponding AUC values were close to 0.5, we still observed stable predictors (defined the same way as for CD-vs.-HC classifier analysis, see section Materials and Methods). However, the number of stable predictors for UC-vs.-HC classifiers (seven taxonomic and four phenotypic) was much lower than for the respective CD-vs.-HC cases (Figures 5C,D).

The PDP analysis for the predictors of UC-vs.-HC classifiers revealed the relationships between the predictors' values and disease probability that were structurally similar to the CD-vs.-HC case (Table 4, Supplementary Figures 4, 5). For taxonomic features, PDP forms for three predictors were sharply decreasing, and, similarly to the CD-vs.-HC analysis, the sharp drop threshold was close to zero predictor value. The *Gemmiger* and *Ruminococcus* genera showed a similar behavior in the CD-vs.-HC classifier. For four taxonomic genera, their PDPs were smoothly increasing (Table 4, Supplementary Figure 4). For the phenotype-based UC-vs.-HC classifier, half of the predictors' PDPs were smoothly increasing, while the other half were smoothly decreasing (Table 4, Supplementary Figure 5).

## Discussion

High-throughput sequencing surveys of the human gut microbiome provide amplicon or WGS sequence datasets that are promising for noninvasive disease risk prediction; however, the task of extracting meaningful biomarkers from such data is still challenging. Machine learning (ML) methods including deep neural networks are powerful for understanding connections of the human gut microbiome to human health (Zhou and Gallins, 2019). During meta-analysis of composition profiles, the corresponding sets of features (e.g., OTUs or ASVs) may be incomparable across different studies due to different laboratory methods/protocols, such as 16S rRNA sequencing region, and quality control (QC) parameters. Traditionally, this problem is solved by aggregating raw ASV (or OTU) features into more biologically meaningful taxonomic features (clade names at the family, genus, or species level). Nevertheless, this aggregation is not universal because it depends on the taxonomic resolution provided by the sequenced gene region. For instance, depending on the ASV length, one might assign unambiguous taxonomic descriptions either up to the species level (*Escherichia coli*) or up to the genus level (*Escherichia*), or even up to the family level (*Enterobacteriaceae*) when the taxonomic resolution is insufficient to distinguish one microorganism from another (e.g., *Escherichia coli* and *Salmonella enterica*). Such gross aggregation leads to the loss of details in phylogenetic description. This is partially remedied through the use of the multi-taxonomic assignment (MTA) approach (see section Materials and Methods), which consists in resolving taxonomic ambiguities not by aggregation on a higher taxonomic level but via listing of all organisms phylogenetically related to the considered ASV (e.g., *Bacteroides ovatus/vulgatus*).

In this study, we introduced yet another approach for feature aggregation, which utilizes the metagenomically predicted metabolic features that are computed for each 16S sample using our previously developed metabolic phenotype profiling tool. The computed CPI values represent the expected fraction of bacterial cells in the community possessing a certain metabolic capability (a phenotype). A collection of CPI values for a selected group of phenotypes, termed Community Phenotype Profile (CPP), therefore constitutes an alternative set of features that can be used for ML. This approach has the following obvious advantages. Firstly, the set of phenotypic features is universal to all organisms and thus can serve as a basis for a cross-study analysis. Secondly, the separation of groups (HC vs. CD/UC in this study) is performed based on the bacterial metabolism and, hence, leads to a straightforward biological interpretation. Thirdly, it allows to refine the classification results in the future when new phenotypic features are added to the CPP. Finally, being based on curated metabolic pathways refined for the species of a specific niche (here, the human gut), our method largely resolves the low-accuracy limitation of many existing metagenomic prediction methods mentioned in the Introduction. However, the limitation related to poor prediction of horizontally transferred genes is not targeted by our approach.

Another problem one deals with in metagenomics (and in ML in general) is high dimensionality of data with low sample count. In ML applications like computer vision, this is solved by the extensive use of data augmentation techniques which allow to drastically enlarge sample count. Unfortunately, they are not applicable to the microbiome studies due to the uniqueness of each sample. Therefore, the only remaining option is the reduction of feature space. Mixing of features by PCA-like methods is not suitable if one wants to preserve biological interpretability; therefore, aggregation of features into taxonomies or CPIs is a preferable choice for dimension reduction. However, in the latter case this aggregation may not be metabolism—but rather phylogeny—driven due to low phylogenetic diversity of organisms possessing a certain phenotype. This implies that the CPI value for such phenotypes would reflect, in the first place, the relative abundance of the phylogenetically narrow group of the phenotype carrier and would obstruct metabolism-driven interpretability. In order to eliminate phenotypic features with low phylogenetic diversity of their contributors from further analysis, we developed and applied a concept of Phenotype Alpha Diversity. Using the calculated PAD values, we filtered out the phenotypes with corresponding contributions to their CPI values coming from phylogenetically narrow groups of organisms, thus retaining phenotypic features which are robust for metabolism-driven inference.

We assessed the applicability of our methodology for differentiating microbiome samples from healthy subjects and patients, using the example of two most prominent IBD conditions, CD and UC, the diseases linked to profound shifts in the microbial community structure. First of all, the selected groups of phenotypes almost completely reflect information about the differences between the microbial communities' taxonomy of healthy people and patients with IBD. The difference in phenotype-based and taxonomy-based classifier performance characteristics (sensitivity, specificity, and AUC) was lower than 0.08 for each of the explored conditions (UC and CD) (Figures 3A, 5A). Thus, we can conclude that despite the limited set of phenotypes selected for the analysis, in the case of IBD they contain most of the information about microbial signature of the investigated diseases.

The second question we wanted to answer was if the phenotypes could introduce new information useful for interpretation of disease relation to microbiome compared to taxonomy. For this purpose, the stable predictors obtained for taxonomic and phenotypic classifiers were compared. The introduction of new information using phenotypes is well demonstrated by the difference in forms and direction of stable predictors PDPs.

The majority of CD taxonomic stable predictors showed negative associations with CD output (Figure 3C). This is consistent with previous observations that IBD condition can be characterized by the depletion of beneficial taxa rather than by the prevalence of pro-inflammatory ones (Duvallet et al., 2017; Wirbel et al., 2020). Such observations were made not only for IBD. More generally, the “Anna Karenina principle” was proposed in application to animal microbiomes: “healthy microbiomes are all alike; each unhealthy microbiome is unhealthy in its own way” (Zaneveld et al., 2017). This principle was proposed for the taxonomic composition of microbiomes. However, it is unknown whether it can be implemented if we consider the functional potential of the community. The majority of CD taxonomic predictors showed a sharply decreasing PDP form with a sharp descent near zero abundance (Supplementary Figure 2). This suggests that the CD condition is associated with the complete absence of these taxa. Among such predictors, there is one of the main primary polysaccharide degraders of the human gut microbiome—*Ruminococcus* (Koropatkin et al., 2012). The primary degradation of fibers is essential for butyrate production. The group also includes other well-known [*Coprococcus* (Pryde et al., 2002; Louis et al., 2014), *Faecalibacterium*] and potential [*Oscillospira* (Gophna et al., 2017)] butyrate producers. *Faecalibacterium* is known for its anti-inflammatory properties, being depleted in the gut of CD patients (Quévrain et al., 2016). Interestingly, the production of butyrate is not thought to be the key anti-inflammatory feature of the bacterium (Sokol et al., 2008; Miquel et al., 2013; Quévrain et al., 2016). It was suggested that *F. prausnitzii* can influence immune response through the production of other metabolites (Sokol et al., 2008; Breyner et al., 2017). Only one taxon, *Flavonifractor*, showed an increasing PDP form. The taxon is known to have proinflammatory properties and was previously shown to be increased in some CD patients (Tyakht et al., 2018).

Unlike the taxonomic predictors for the CD classifier, the majority of phenotypic predictors show increasing PDP forms (Supplementary Figure 3). It can be suggested that each increasing phenotypic predictor is a functional representation of few increasing taxonomic predictors. However, the filter applied to the diversity of phenotypes excludes the use of phenotypes represented in a small number of taxa. Thus, we can speculate that despite the fact that the taxonomic signature is composed mainly of commensal taxa, functionally the microbiome of patients is characterized to a greater extent by pro-inflammatory predictors. In this case, we see that the “Anna Karenina principle” for taxonomic composition of the microbiome (Zaneveld et al., 2017) is not fulfilled for its functional potential. It was previously shown that a healthy gut microbial community is characterized by functional homeostasis. This means that despite the differences in the taxonomic composition of different people communities, the metabolic potential of these communities is quite similar (Eng and Borenstein, 2018). Our observations in the case of Crohn's disease support this concept. Taxonomically, the imbalance included the increase of various taxa in different individuals, but functionally, we see the increase of the same functions. Thus, the application of microbial phenotypes allowed us to identify universal markers qualitatively different from taxonomic ones and to provide a new layer of information for further interpretations.

The phenotypic stable predictors positively associated with CD were generally linked to degradation of host-derived carbohydrates (Figure 3D). Firstly, it was evident at the level of specific families of glycoside hydrolases; thus, we observed an increase of microbial community representation of the GH38, GH18, and GH125 families involved in catabolism of N-linked glycans that are constituents of the host mucus (El Kaoutari et al., 2013; Engevik et al., 2019). Another family of glycosyl hydrolases with increased representation in CD samples, GH29, includes exo-acting α-fucosidases that are involved in the degradation of O-linked glycans, in particular mucin (Tailford et al., 2015). In particular, GH29 is present in the mucin-dwelling bacteria from the genera *Ruminococcus* (Crost et al., 2013) and *Akkermansia* (El Kaoutari et al., 2013). These changes were reflected by the increased propensity toward utilization of five monosaccharides including neuraminic acid (NANa), N-acetylglucosamine (GlcNAc), mannose (Man), and glucose (Glc) that constitute O- and N-linked glycans, as well as ribose (Rib). The latter monosaccharide is utilized by many gut bacteria such as *Bacteroides* spp., while ribose and ribose-containing molecules such as nucleic acids may serve as nutrients for these gut symbionts (Glowacki et al., 2020). The increased potential of mannose metabolism was reported in patients with ileal CD (Morgan et al., 2012). The list of the phenotypes increased in CD samples also included histidine (His) amino acid degradation—apparently, reflecting the high availability of host-derived amino acids from the inflamed tissue. Further, the increased vitamin B7 (biotin) synthesis potential was in line with the reported upregulation of the respective biosynthetic enzymes in stool metatranscriptomes of IBD patients (Das et al., 2019). Overall, these observations suggest that the CD-associated microbiome is prominent by its ability to degrade the mucus, apparently due to highly inflammatory milieu and excessive shedding of intestinal epithelial cells (Png et al., 2010; Blander, 2016). Another phenotypic feature increased in CD was propionate production potential. Although considered to be anti-inflammatory as butyrate (Tedelind et al., 2007), some of its effects on the immune cells are opposite to the ones of butyrate (Cavaglieri et al., 2003). Recently, propionate was shown to promote the virulent properties of CD-associated *Escherichia coli* (Ormsby et al., 2020; Pobeguts et al., 2020), the key taxon linked to the disease in previous studies.

On the other hand, there were only a few phenotypic stable features negatively associated with CD, including the butyrate production, histidine biosynthesis, and the GH94 family of glycoside hydrolases (Figure 3D). The butyrate is one of the most studied beneficial microbiome-derived metabolites with anti-inflammatory potential. First of all, it serves as an energy source for the colonic epithelium, therefore preventing mucosal atrophy (Hamer et al., 2008). In addition, butyrate possesses some direct immuno-modulatory effects like suppression of nuclear factor kappa B (NF-κB) activation (Hamer et al., 2008), signaling through G-protein-coupled receptors (Hamer et al., 2008) and GPR109A (Singh et al., 2014). Interestingly, among the stable predictors of CD, most taxa known as butyrate producers manifested sharp PDP form, while the butyrate production phenotype CPI was smooth—apparently reflecting the averaging of their contributions. Depletion of the histidine synthesis potential in CD samples is in line with the abovementioned increase of the histidine degradation phenotype. Strikingly, unlike GH families positively associated with CD that were mainly involved in mucus degradation, the GH94 family phosphorylases that cleave β-glycosidic bonds in cellobiose and cellodextrin are involved in plant cell wall degradation (Cantarel et al., 2012).

Ulcerative colitis, the second major IBD condition we investigated, is characterized by a less pronounced microbiome disruption compared with the Crohn's disease—resulting in a generally lower classification performance. It is in line with the previous reports (Halfvarson et al., 2017; Imhann et al., 2018; Franzosa et al., 2019; Clooney et al., 2020). The reason for this can be the different epidemiological and clinicopathological pictures of the diseases. In terms of epidemiology, it was hypothesized that in Crohn's disease etiology, the early-life abnormal cross-talk between microbiome and immune system plays the essential role, while in ulcerative colitis it is dysbiosis that occurred at any time of life (Beaugerie et al., 2018). In terms of clinical picture differences, in ulcerative colitis inflammation foci are located in rectum and colon, while Crohn's disease can also involve the upper parts of the gastrointestinal tract. IBD location was shown to affect dysbiosis pictures of the diseases (Imhann et al., 2018).

The UC classifiers demonstrated generally lower performance compared to the CD and produced fewer stable predictors, most of which are shared with CD predictors. In hand with the lower performance of the UC-vs.-HC classifier, the identified stable predictors were also less reliable compared to the CD stable predictors. Three taxonomic genera, *Gemmiger, Flavonifractor*, and *Ruminococcus*, are shared between the CD and UC predictors. Among microbial genera that are specific for the UC-vs.-HC classifier, there were the mucin-degrading *Akkermansia* with sharply decreasing PDP form, and three taxa with smoothly increasing PDP form which are *Streptococcus, Oscillibacter*, and *Flintibacter*. Mucolytic taxa were previously shown to be enriched in the intestinal epithelium of IBD patients compared to healthy controls (Png et al., 2010), with the only exception for *Akkermansia*, which had significantly higher abundance in control samples, in line with our findings. Several other studies showed the protective role of *Akkermansia* in IBD (Bian et al., 2019; Earley et al., 2019). Interestingly, neither the butyrate-producing taxa nor the phenotype of butyrate synthesis itself were detected among the stable predictors for UC. It resonates with the previous studies showing that microbiome butyrate-synthetic capacity was reduced only in patients with active UC, but not in ones with inactive stage of disease, while for Crohn's disease, the association was observed for both stages (Laserna-Mendieta et al., 2018). The list of phenotypic stable predictors positively associated with UC is shorter than the respective list for the CD and overlaps with the latter by including the mannose utilization (Man) and the glycoside hydrolase family GH125 of exo-mannosidases involved in N-glycan degradation. However, while for the CD we observed a sharply increasing PDP form for Man (with the threshold around 0.2–0.3 phenotype abundance), for the UC the form was smoothly increasing. The decrease of histidine (His) and arginine (Arg) amino acid synthesis potentials in UC samples is likely linked to a higher abundance of free amino acids originating from the inflamed host tissue in the gut.

We discovered that the classification performance varies across different studies. A classifier trained on one geographic population might be not that precise for a cohort from another country. Besides possible technological differences, this effect could be contributed to by the geography-specific features not only of the healthy microbiome composition but also in the patients with diseases. Similar effects were reported before, e.g., for type 2 diabetes (Karlsson et al., 2013). Further extended regional multicenter studies with large cohorts of healthy and affected subjects are required to elucidate the universal character of microbial phenotypes' robustness and concordance with the taxonomic features.

## Conclusions

We developed a novel computational approach that uses a concept of metabolic phenotypes toward the microbiome-based classification of clinical status and assessed its performance using 16S amplicon sequencing data from multiple IBD studies. Although the set of assessed metabolic functions and pathways was limited to metabolism of sugars (including GH enzymes involved in polysaccharide degradation and SCFA production pathways), amino acids, and vitamins, our results suggest that the performance of the microbial phenotype-based classification was comparable to the state-of-art taxonomic approach.

Feature design in machine learning algorithms, which is based on cumulative metabolic potential of microbiome species (estimated via CPIs), can provide additional functional insights on the deviations of microbiome from homeostasis in disease. To provide truly metabolism-driven inference, these metabolic features are likely to account for the collective action of a phylogenetically diverse community of a particular phenotype carrier, which is reflected in the Phenotype Alpha Diversity (PAD) metric. In CD, while the community structure was characterized by the depletion of many commensal taxa rather than the presence of specific opportunists, the functional imbalance was revealed as an enrichment of inflammation-related phenotypes not reflected at the taxonomic level.

The major indicators of functional imbalance of microbiome in IBD reflect the adaptation to the inflammatory environment by including increased potential for degradation of mucin-derived carbohydrates and amino acids and propionate synthesis, while the healthy gut is characterized by enriched degradation of dietary complex carbohydrates and synthesis of butyrate and amino acids. Analysis of the abundance-dependent contribution of each feature to the classification outcome using PDP suggests that the presence of most taxa negatively associated with IBD is more important than their abundance. Further, the PDP patterns reflect how a plethora of taxa (each showing sharply decreasing PDP form in IBD) can functionally “convolve” into a single phenotype (with smoothly decreasing PDP), as exemplified by the case of butyrate producers.

Our results show that the analysis based on microbial phenotypes can provide interpretable insights into the host–microbiome mechanisms of disease. Extension of the phenotype list to include metabolism of specific polysaccharides, lipids, and bile acids will provide further insights into the possible mechanisms of gut microbiome metabolic contribution to the risks and onset and development of the disease. Further expansion of the reference microbial genomes database with predicted metabolic phenotypes will allow one to apply the phenotype profiling approach to microbial communities of other human body sites, and more generally to various environmental and industrial microbiomes.

## Data Availability Statement

Publicly available datasets were analyzed in this study. This data can be found here: www.ebi.ac.uk/ena: project IDs PRJNA422193 and PRJEB22028, ega-archive.org: project IDs EGAS00001002702 and EGAS00001001704.

## Author Contributions

AT and DR conceived and designed the research project. TS performed primary analysis of the sequencing data. SI, PN, and DR developed CPI/MTA/PAD concepts and analyzed metabolic phenotypes. NK, SI, DE, and AT performed machine learning analysis of the obtained taxonomic and metabolic phenotype profiles. NK, SI, DR, and AT wrote the manuscript. All authors read and approved the final manuscript.

## Conflict of Interest

PN was employed by the company PhenoBiome Inc.; AT, NK, DE, and TS were employed by the company Atlas Biomed Group—Knomics LLC. DR and PN are co-founders of PhenoBiome Inc. The remaining author declares that the research was conducted in the absence of any commercial or financial relationships that could be construed as a potential conflict of interest.
