# Supplementary material for: Metabolic Phenotypes as Potential Biomarkers for Linking Gut Microbiome With Inflammatory Bowel Diseases
Source: Front Mol Biosci. 2021 Jan 18;7:603740. doi: 10.3389/fmolb.2020.603740 (PMC7848230; doi:10.3389/fmolb.2020.603740)
Supplement: Supplementary file 4 [file Image_1.PDF]

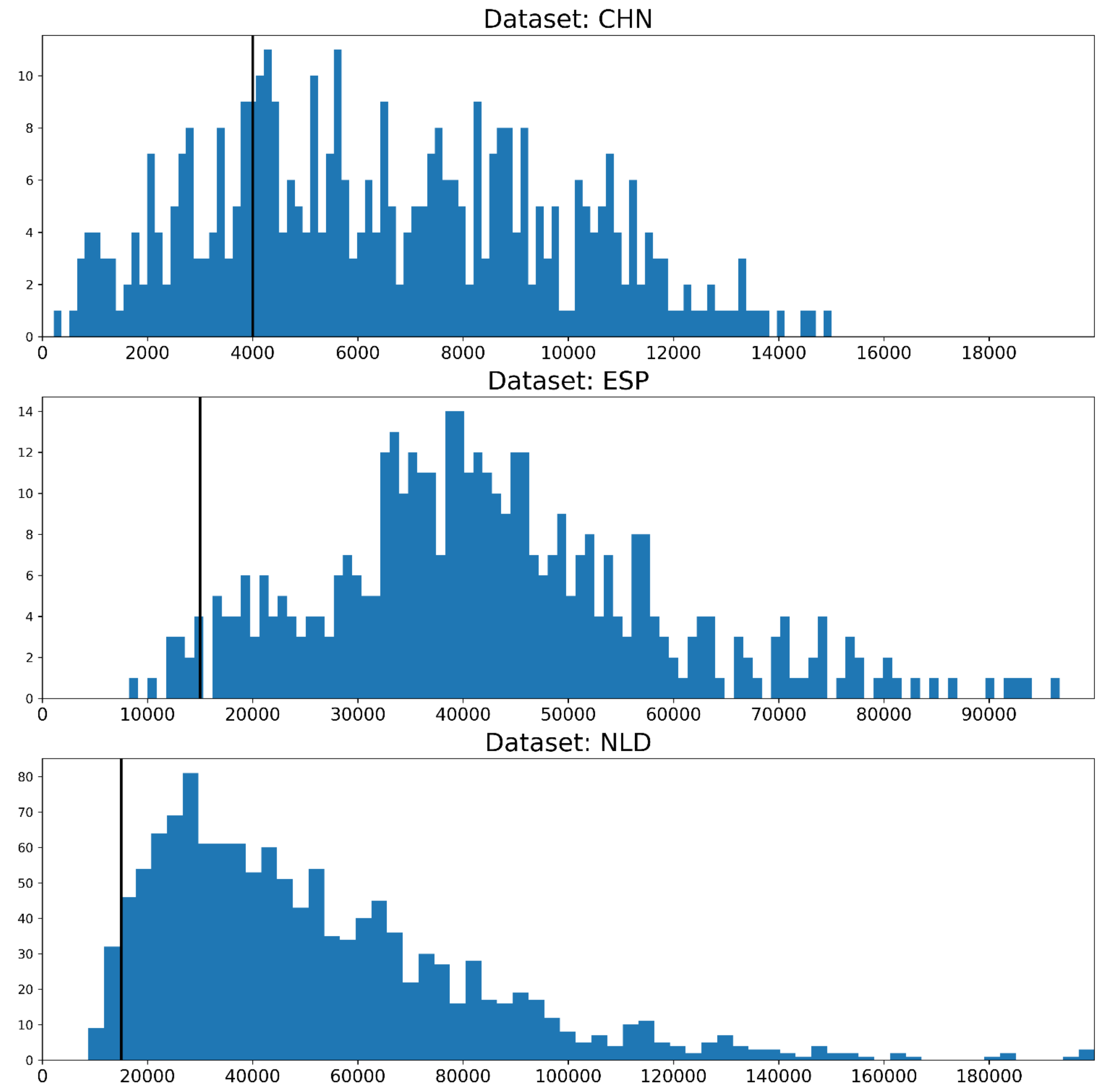

Figure S1. Reads number distribution for samples in analyzed datasets. Black vertical lines show coverage thresholds below which samples were dropped from consideration due to low read count: 4000 reads for CHN, 15000 reads for ESP and NLD.
