# Supplementary material for: Metabolic Phenotypes as Potential Biomarkers for Linking Gut Microbiome With Inflammatory Bowel Diseases
Source: Front Mol Biosci. 2021 Jan 18;7:603740. doi: 10.3389/fmolb.2020.603740 (PMC7848230; doi:10.3389/fmolb.2020.603740)
Supplement: Supplementary file 6 [file Image_3.PDF]

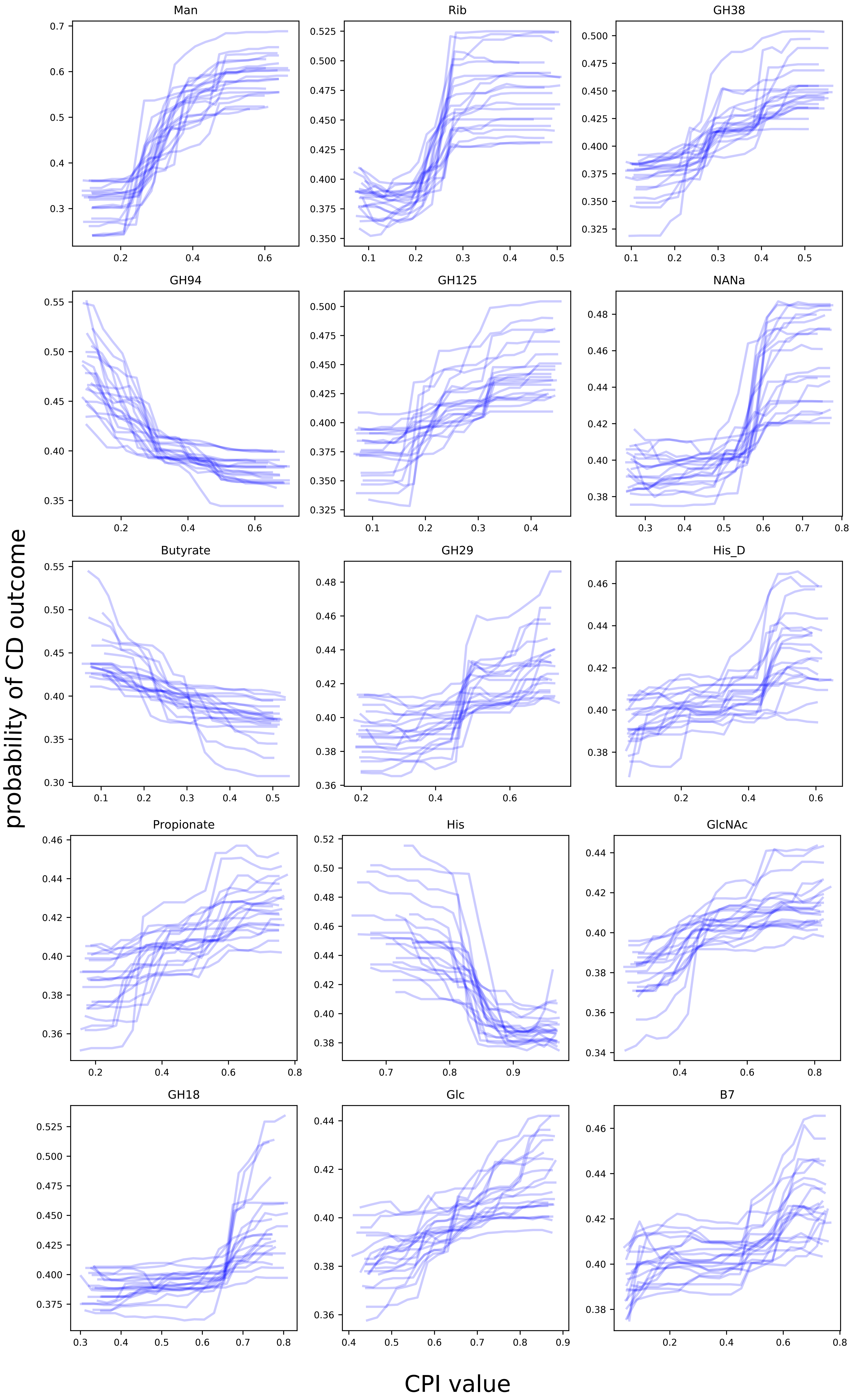

Figure S3. Partial dependence plots for phenotypic stable predictors and CD outcome. The x axis of the plots denotes CPI values and y axis - the probability of CD classification outcome.
