# Supplementary material for: Metabolic Phenotypes as Potential Biomarkers for Linking Gut Microbiome With Inflammatory Bowel Diseases
Source: Front Mol Biosci. 2021 Jan 18;7:603740. doi: 10.3389/fmolb.2020.603740 (PMC7848230; doi:10.3389/fmolb.2020.603740)
Supplement: Supplementary file 7 [file Image_4.PDF]

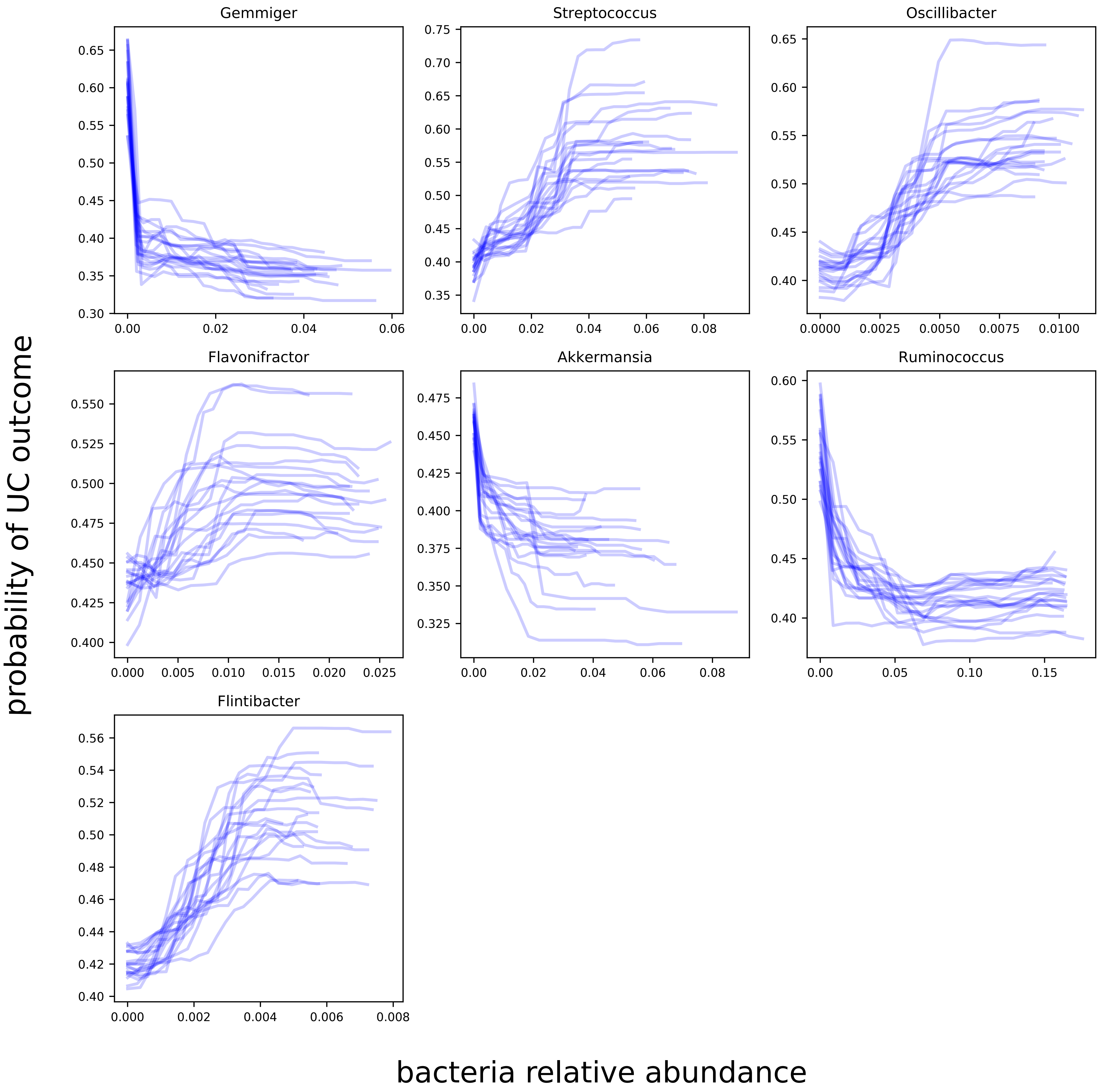

Figure S4. Partial dependence plots for taxonomic stable predictors and UC outcome.The x axis of the plots denotes bacteria abundance and y axis - the probability of UC classification outcome.
